# A cross sectional assessment of health related quality of life among patients with Hepatitis-B in Pakistan

**DOI:** 10.1186/1477-7525-10-91

**Published:** 2012-08-06

**Authors:** Noman ul Haq, Mohamed Azmi Hassali, Asrul A Shafie, Fahad Saleem, Hisham Aljadhey

**Affiliations:** 1 Department of Pharmacy, University of Baluchistan, Quetta, Pakistan/Discipline of Social and Administrative Pharmacy, School of Pharmaceutical Sciences, Universiti Sains Malaysia, Penang, Malaysia; 2 Discipline of Social and Administrative Pharmacy, School of Pharmaceutical Sciences, Universiti Sains Malaysia, Penang, Malaysia; 3 College of Pharmacy, King Saud University, Riyadh, Saudi Arabia

**Keywords:** Health Related Quality of Life, Hepatitis B, Euroqol EQ-5D, Pakistan

## Abstract

**Objective:**

The study aims to assess Health Related Quality of Life (HRQoL) among Hepatitis B (HB) patients and to identify significant predictors of the HRQoL in HB patients of Quetta, Pakistan.

**Methods:**

A cross sectional study by adopting European Quality of Life scale (EQ-5D) for the assessment of HRQoL was conducted. All registered HB patients attending two public hospitals in Quetta, Pakistan were approached for study. Descriptive statistics were used to describe demographic and disease related characteristics of the patients. HRQoL was scored using values adapted from the United Kingdom general population survey. EQ-5D scale scores were compared with Mann–Whitney and Kruskal-Wallis test. Standard multiple regression analysis was performed to identify predictors of HRQoL. All analyses were performed using SPSS v 16.0.

**Results:**

Three hundred and ninety HB patients were enrolled in the study. Majority of the participants (n = 126, 32.3%) were categorized in the age group of 18-27 years (36.07 ± 9.23). HRQoL was measured as poor in the current study patients (0.3498 ± 0.31785). The multivariate analysis revealed a significant model (F_10, 380_ = 40.04, P < 0.001, adjusted r^2^ = 0.401). Educational level (β = 0.399, p = 0.025) emerged as a positive predictor of HRQoL. Age, gender, occupation, income and locality were not predictive of better quality of life in HB patients.

**Conclusions:**

Hepatitis B has an adverse affect on patients’ well-being and over all HRQoL. The study findings implicate the need of health promotion among HB patients. Improving the educational status and imparting disease related information for the local population can results in better control and management of HB.

## Background

Quality of life (QOL) includes subjective evaluation of positive and negative aspects of life [1]. It is an individuals’ perception of their position in life within the context of the culture and value systems in relation to their goals, expectations, standards, and concerns [2]. On the contrary, Health Related Quality of Life (HRQoL) and its determinants encompass aspects of overall quality of life that affect health (physical or mental) [3-6]. Therefore, compared to QOL, HRQoL is an important tool in identifying patient's perception of being ill and the assessment of treatment outcomes [7].

Hepatitis-B (HB) is one of the most common liver infections in the world. More than 2 billion people have been infected by HB worldwide, and out of those, 350 million have chronic, lifelong infection. An estimated 0.6 million people die each year from HB-related liver diseases and 3–4 million people are newly infected [8,9]. The development of chronic conditions with decreased life expectancies is very disturbing for the patients [10]. The advance stage development (liver cirrhosis and hepatocellular carcinoma), expensive treatments and fear of death associated with HB, affects patients’ daily life activities and results in decrease health status [11,12]. In addition, patients with HB often report decreased HRQoL because of fatigue, loss of self-esteem, inability to function at work, anxiety, depression, and other emotional problems [13].

Shifting the concerns towards HRQoL and developing countries, the very concept is often neglected when patients are treated for chronic diseases like HB. Within this context, Pakistan being one of the highest populated countries in the world has more than 24% of the population living below the national poverty line [14]. Lack of health facilities and human recourses in health sector is counted as a major obstacle in delivering optimal health care to the population. In addition, uncaring inhuman behaviour and unavailability of the doctors is another major concern [15]. In the presence of such entities, the healthcare is unable to provide the ‘required’ facilities and in return affects the health status of the patients.

To the best of our knowledge, little is known about the HRQoL status among Pakistani population suffering from HB. Although few studies [16-18] reported HRQoL among Pakistani population suffering from multiple liver diseases, there is paucity of data concerning HRQoL solely among HB patients. Therefore, this study aims to evaluate the profile and predictors of HRQoL among HB patients attending public hospitals in Quetta city, Pakistan.

## Methods

### Study design, settings and sampling

A questionnaire based, cross sectional analysis was conducted. Registered patients from two public hospitals (Sandmen Provisional Hospital and Bolan Medical Complex Hospital) of Quetta city, Pakistan were included for the study. Both of these hospitals are tertiary care institutes and being public in nature provide treatment to the majority of the population.

HB is reported to affect 11% of population in Pakistan [19,20]. Therefore, a prevalence based sample of 390 HB patients was selected for the study from March 2011 to July 2011 [21,22]. Patients aging 18 years and above, having confirmed diagnosis of HB, and familiar with Urdu (National language of Pakistan) were included in the study. Patients having co-morbidities, immigrants from other countries and pregnant ladies were excluded.

## Ethical approval

This study was performed according to the ethical standards for human experimentation [23]. The Joint Clinical Research Committee (for Sandmen Provisional Hospital and Bolan Medical Complex Hospital) approved the study protocol (No.EA/NUH/1205-2009). Written consent was also taken from the patients prior to data collection. Patients were made sure about the confidentiality of their responses and their right to withdraw from the study.

### Study instrument

European Quality of Life scale EQ-5D was used to measure HRQoL. EQ-5D is a standardized generic HRQoL instrument developed by the EuroQoL group. It provides a simple descriptive summary and a single index value for health status [24]. EQ-5D consists of five domains (i.e. mobility, self-care, usual activities, pain/discomfort, and anxiety/depression) each of which can further categorized into three levels of severity (no problems/some or moderate problems/extreme problems). Two hundred and twenty six different health responses can be achieved describing health status of respondents. VAS (Visual analogue scale) is the other portion of EQ-5D consisting of a 20 cm health meter with two distinct end points (i.e. 100 which is the best imaginable health state and 0 which is the worst imaginable health state). It is a valid, easy to administer, less time consuming instrument which is available in Urdu [25]. EQ-5D is a self-administered instrument but six pharmacists were recruited and trained by the researcher team, to help patients having difficulty in understanding the questions. This study was registered with EuroQoL. The internal consistency and validity of questionnaire was ensured (the Cronbach’s alpha value being 0.65 for the instrument used in the study) [26].

### Statistical analysis

Descriptive analysis of patients’ demographic information was performed. Categorical variables were measured as percentages while continuous variables were expressed as mean ± standard deviation. As general population norms for Pakistani population are not documented, EQ-5D was scored by using values derived from the UK general population survey reported in 1995 [27]. Mann–Whitney and Kruskal Wallis tests were used as Kolmogrov-Smirnov test revealed non normal distribution of the data. Standard multivariate regression analysis was applied to investigate the effects of demographic variables on HRQoL in the current cohort of HB patients. A statistical value of P < 0.05 was taken as significant. All analyses were performed using SPSS 16.0 (SPSS Inc., Chicago, IL).

## Results

### Demographic characteristics

Table 1 describes the demographic information of the study participants. Mean age of respondents was 36.07 ± 9.23 years and the cohort was dominated by 232 (59.5%) of males. One hundred and four (26.7%) had primary level of education. One hundred and sixty two (41.55%) were unemployed with 151 (38.7%) having no income. Two hundred and seventy three (70%) were having urban residency.

**Table 1:** Demographic Characteristics of study respondents (n = 390)

**Description**	**Frequency (390)**	**Percentage**
***Age (39.02*** **±** ***9.23) years***		
18-27	85	21.8
28-37	125	32.1
38-47	136	34.9
48-57	35	9.0
58 < year	9	2.3
***Gender***		
Male	232	59.5
Female	158	40.5
***Education***		
Illiterate	19	4.8
Religious Education	67	17.2
Primary	104	26.7
Metric	54	13.8
Intermediate	67	17.2
Graduation	55	14.1
Post-Graduation	24	6.2
***Occupation***		
Unemployed	162	41.5
Government Servant	33	8.5
Private Servant	111	28.5
Self Employed	84	21.5
***Income***		
No Income *	151	38.7
< Pak Rs. 5000	51	13.1
5001-10000	36	9.1
10001-15000	81	20.8
>15001	71	18.2
***Locality***		
Urban	273	70.0
Rural	117	30.0

*1 PKR = 0.0115527 USD.

### EQ-5D health status

A total of 41 health states were reported by the patients. Poor HRQoL was measured as reported mean EQ-5D descriptive score and EQ-VAS score were 0.37 ± 0.30 and 57.12 ± 10.9 respectively. Sixty three (16.15%) reported some problem in the first, third, fourth and fifth domain, whereas no problem in the second domain as shown in Table 2.

**Table 2:** self-reported (EQ-5D) Health States

**S No**	**EQ-5D**	**Frequency**	**Percentage**
1	11212	10	2.56
2	11222	11	2.82
3	12222	8	2.05
4	12321	11	2.82
5	12322	15	3.85
6	12323	8	2.05
7	12331	18	4.62
8	13223	1	0.26
9	21121	57	14.62
10	21122	8	2.05
11	21123	4	1.03
12	21132	3	0.77
13	21221	5	1.28
14	21222	63	16.15
15	21223	15	3.85
16	21232	8	2.05
17	21233	4	1.03
18	21312	8	2.05
19	21323	7	1.79
20	21332	5	1.28
21	21333	1	0.26
22	22111	1	0.26
23	22112	1	0.26
24	22113	4	1.03
25	22122	10	0.56
26	22211	4	1.03
27	22222	9	2.31
28	22232	6	1.54
29	22322	5	1.28
30	22333	9	2.31
31	23113	5	1.28
32	23122	4	1.03
33	23123	8	2.05
34	23212	7	1.79
35	23222	6	1.54
36	23223	5	1.28
37	23233	4	1.03
38	23322	8	2.05
39	31222	5	1.28
40	32113	6	1.54
41	32222	13	3.33

Two hundred and eighty three (72.6%) participants indicated some problem in first domain (Mobility), 215 (55.1%) indicated no problem in second domain (self-care), 186 (47.7%) indicated some problems in third domain (Usual Work), 288 (73.8%) indicated some pain and discomfort in fourth domain (Pain and Discomfort) and 213 (54.6%) reported moderate anxiety and depression in the fifth domain (Anxiety and Depression) as shown in Table 3.

**Table 3:** EQ-5D Domains

**EQ-5D Domain**	**Frequency**	**Percentage**
***First Domain (Mobility)***		
No Problem in walking about	82	21.0
Some Problem in Walking about	283	72.6
Confined to bed	25	6.4
***Second Domain (Self-care)***		
No Problem in self care	215	55.1
Some Problem in washing and dressing myself	127	32.6
wash and dress myself	48	12.3
***Third Domain (Usual Work)***		
No Problem in performing usual activities	110	28.2
Some Problems in performing usual activities	186	47.7
Unable to perform usual activities	48	24.1
***Forth Domain (Pain and Discomfort)***		
No pain and discomfort	45	11.5
Some pain and discomfort	288	73.8
Extreme pain and discomfort	57	14.6
***Fifth Domain (Anxiety and Depression)***		
Not anxious or depress	97	24.9
Moderately anxious or depress	213	54.6
Extremely anxious or depress	80	20.5

Only gender was found to significantly associated with VAS score (*p = 0.014*), (male 58.3 ± 10.692 and female 55.58 ± 11.002), however, there was no significant different between HRQoL and other study variables as described in Table 4 and 5.

**Table 4:** Mean EQ-5D scores

**Description**	**N**	**Mean EQ5D**	**Std**	***p *Value**
		**Score**	**Deviation**	
Age* (36.62 **±** ***9.597)***				
18-27	85	0.3811	0.29440	
28-37	125	0.3775	0.29613	
38-47	136	0.3925	0.29432	*0.056*
48-57	35	0.2503	0.34559	
58 < year	9			
Gender**				
Male	232	0.3636	0.31061	*0.584*
Female	158	0.3850	0.28596	
Education*				
Illiterate	19	0.3178	0.28567	
Religious Only	67	0.3609	0.30195	
Primary	104	0.3731	0.31979	
Metric	54	0.4130	0.27255	*0.613*
Intermediate	67	0.3287	0.30961	
Graduation	55	0.3812	0.29382	
Post-Graduation	24	0.4517	0.27869	
Occupation*				
Unemployed	162	0.3849	0.29687	
Government Servant	33	0.4340	0.32251	*0.521*
Private Servant	111	0.3472	0.29438	
Self Employed	84	0.3565	0.30805	
Income*				
Nil	151	0.3876	0.29377	
< Pak Rs. 5000	51	0.3360	0.32486	
5001-10000	36	0.3889	0.29434	*0.652*
10001-15000	81	0.3565	0.30586	
>15001	71	0.3752	0.29992	
Locality**				
Urban	273	0.3751	0.30002	*0.795*
Rural	117	0.3655	0.30339	
**Total**	**390**	**0.3722**	**0.30068**	

* Kruskal Wallis Test.

** Mann Whitney Test.

**Table 5:** Mean VAS scores

**Description**	**N**	**Mean**	**Std**	***p *Value**
		**EQ VAS**	**Deviation**	
Age* (39.02 **±** ***9.2)***				
18-27	85	56.9	11.406	*0.291*
28-37	125	57.4	10.475	
38-47	136	58.0	10.953	
48-57	35	54.2	9.997	
58 < year	9	53.2	10.215	
Gender**				
Male	232	58.3	10.692	***0.014***
Female	158	55.5	11.002	
Education*				
Illiterate	19	53.8	8.719	*0.112*
Religious Only	67	55.4	10.418	
Primary	104	56.4	11.525	
Metric (SSC)	54	58.3	10.907	
Intermediate (HSC)	67	58.6	10.321	
Graduation	55	59.3	9.438	
Post-Graduation	24	56.2	14.441	
Occupation*				
Unemployed	162	56.5	10.591	*0.274*
Government Servant	33	56.4	9.584	
Private Servant	111	59.0	11.186	
Self Employed	84	56.4	9.819	
Income*				
No Income	151	56.4	10.962	*0.838*
< Pak Rs. 5000	51	56.4	12.025	
5001-10000	36	57.4	11.126	
10001-15000	81	58.8	10.774	
>15001	71	57.3	9.991	
Locality**				
Urban	273	57.6	10.906	*0.227*
Rural	117	56.2	10.831	
**Total**	**390**	**57.2**	**10.888**	

Table 6 highlights the results of the multiple regression analysis. Using the enter method, a significant model emerged (F_*10, 380*_ = 40.04, P < 0.001, adjusted r^2^ = 0.401). Educational level emerged as the influencing factors on HRQoL. The multiple regression analysis also found that age, gender, occupation, income and locality were not significantly associated with HRQoL.

**Table 6:** Multivariate association between study variables and HRQoL

**Predictor Variable**	**Beta**	**P-Value**
Age	0.011	0.566
Gender	-0.025	0.721
Education	0.399	***0.025***
Occupation	0.043	0.470
Income	0.010	0.551
Locality	0.009	0.241

## Discussion

The current study reveals poor HRQoL in HB patients. In addition, the descriptive score was even less than the perceived health status enlightening that actual health condition is even worse than what was perceived by the patients. Awan *et al* from their study conducted in Sargodha, Punjab, Pakistan reported that HRQoL among HB is poor with no relation to the demographic and disease characteristics [17]. The findings were again supported by Atiq *et al* in their study concerning HRQoL in Islamabad, Pakistan [16].

The current study findings are also inline to what is reported in studies from other part of the world. WU *et al* in China reported lower HRQoL in HB patients in both physical function and mental health [28]. Whereas, Tan *et al* stated HB patients had no impairment in physical and mental health, even though there was a significant decrease in HRQoL [29]. Reduced HRQoL in comparison to a healthy population was observed by Svirtlih *et al* in Serbia [12]. A number of studies conducted in United States of America reported that HB attribute to negative physical, social and psychological health status even in absence of severe liver damage [30-32]. Moreover, in a multination survey conducted in United States, Canada, United Kingdom, Spain, Hong Kong, and mainland China by Levy *et al* accounted HB to reduce HRQoL in HB patients with strong impact on HRQoL as the disease progresses [33].

HRQoL had significant relationship with gender in our study. There are mixed results when our findings are compared with studies of same nature. Olson *et al* reported that less physical activities, alcohol use, depression and gender (female) independently influence HRQoL [34]. Sobhonslidsuk *et al* concluded that advance stages of disease, old age, gender (female), low socioeconomic status and financial burden were important factors that reduce HRQoL in HB patients [11]. Goins *et al* concluded that age, sex, education, annual household income, employment status, disease status, and obesity were significant to HRQoL [35]. Lam *et al* reported advanced stage of HB, bilirubin level, psychological co morbidity, younger age and gender (female) were associated with poorer HRQoL [36]. On the contrary, age, disease severity, depression, financial hindrance and threat of death were reported to negatively affect HRQoL in HB patients [17]. Pappa *et al* highlighted age as the only factor that had significant relationship with HRQoL [37]. Younossi *et al* concluded that lower HRQoL in HB patients is independent to all demographic characteristic (including the gender) of the respondents [38].

In literature, the association between education and HRQoL in chronic diseases is well known and persistent [39,40]. In addition, significant results are presented between more and less educated groups [41]. Education is responsible in providing a wide range of utilitarian possessions to the individual that are used to his/her health advantage. Education also develops interest and involvement of patients in improving one's own health which is a key determinant of a successful medical treatment. It is a common observation that better educated people are less likely to develop chronic conditions, or are often in the “controlled” status. In addition to pharmacotherapy, better educated are more likely to adapt life style modification and preventive measures which results in an improvement of HRQoL. Cutler and Muney did report that an additional four years of education lowers five-year mortality by 1.8 percentage points, reduces the risk of heart disease by 2.16 and the risk of diabetes by 1.3 percentage points [42]. The same applied to both developed and developing countries worldwide where more educated were reported to live longer with better health conditions and status [42].

Keeping in view the treatment pattern and time period of chronic illnesses, HB requires lifelong treatment. Developing countries do face a number of challenges in providing optimal health care to all of its population. In Pakistan, majority of healthcare costs are paid by patients themselves, the cost of health care for chronic diseases puts a significant strain on household budgets. Being extremely expensive, people are pushed into poverty because they have to pay directly for health services thus decreasing their HRQoL [25]. In addition, lack of basic health facilities and resources, behavioral aspects and practices influence the patient in real-life scenario. In return, a large number of patients tend to move to other healthcare providers prior to consulting certified practitioners. Prevalence of such entities affects the HRQoL to more extent than it is believed and often results in the development of resistance, hence increasing the cost of therapies and decreasing the HRQoL.

## Conclusion

HB has an adverse impact of patients’ well-being and HRQoL. This study provides baseline assessment for the health status of HB patients and the results could be applied in clinical practice, particularly in early treatment of HB and improving HRQoL. The study findings implicate the need of health promotion among HB patients. Improving the educational status and imparting disease related information for the local population can results in better control and management of HB.

### Limitations

The study is as a cross sectional study on outpatients in public hospitals that are usually approached by low to middle income population. Whereas, the high income group usually uses these facilities in emergency only. Hence the results of our research may not represent the entire population.

## Competing interests

The authors declare that they have no competing interests'.

## Authors’ contribution

NH and FS conducted the survey and drafted the initial manuscript. MAH and AAS designed and supervised the study. HA helped in statistical analysis, interpretation and manuscript revision. All authors read and approved the final manuscript.

## Funding

No funding was received for this study.
